# A process account of the uncontrolled manifold structure of joint space variance in pointing movements

**DOI:** 10.1007/s00422-019-00794-w

**Published:** 2019-02-15

**Authors:** Valère Martin, Hendrik Reimann, Gregor Schöner

**Affiliations:** 1 Institute for Neural Computation, Ruhr-University, Bochum, Germany; 2 Present Address: Bern University of Applied Sciences, Länggasse 85, 3052 Zollikofen, Switzerland; 3 Department of Kinesiology and Applied Physiology, University of Delaware, Newark, DE USA

**Keywords:** Human motor control, Pointing movement, Redundancy, Kinematics, Dynamical systems, Uncontrolled manifold

## Abstract

In many situations, the human movement system has more degrees of freedom than needed to achieve a given movement task. Martin et al. (Neural Comput 21(5):1371–1414, 2009) accounted for signatures of such redundancy like self-motion and motor equivalence in a process model in which a neural oscillator generated timed end-effector virtual trajectories that a neural dynamics transformed into joint virtual trajectories while decoupling task-relevant and task-irrelevant combinations of joint angles. Neural control of muscle activation and the biomechanical dynamics of the arm were taken into account. The model did not address the main signature of redundancy, however, the UCM structure of variance: Many experimental studies have shown that across repetitions, variance of joint configuration trajectories is structured. Combinations of joint angles that affect task variables (lying in the uncontrolled manifold, UCM) are much more variable than combinations of joint angles that do not. This finding has been robust across movement systems, age, and tasks and is often preserved in clinical populations as well. Here, we provide an account for the UCM structure of variance by adding four types of noise sources to the model of Martin et al. (Neural Comput 21(5):1371–1414, 2009). Comparing the model to human pointing movements and systematically examining the role of each noise source and mechanism, we identify three causes of the UCM effect, all of which, we argue, contribute: (1) the decoupling of motor commands across the task-relevant and task-irrelevant subspaces together with “neural” noise at the level of these motor commands; (2) “muscle noise” combined with imperfect control of the limb; (3) back-coupling of sensed joint configurations into the motor commands which then yield to the sensed joint configuration within the UCM.

## Introduction

How are the many kinematic degrees of freedom (DoF) of the human motor system and the muscles that actuate them harnessed to achieve a particular movement goal? This is the classical “degree of freedom problem” that has been a major theme in motor control at least since Bernstein (1967). The problem arises because motor systems are redundant (or abundant, see Gelfand and Latash 1998) for many tasks; that is, there are typically more joints or muscles available than needed to control a set of task variables like the position or orientation of the hand in space.

It is not clear, a priori, that this is a problem for the central nervous system (CNS) because, in principle, the neural networks that generate and control movement could simply be structured such that they provide one particular solution among the many possible ones. It would then be merely a problem for us, the scientists, to find out which solution that is. But movement scientists nourish a long-standing intuition and hypothesis that the DoF problem is a problem for the CNS. This intuition is based on empirical evidence that the CNS is flexible about which particular solution is employed under different conditions. Bernstein famously observed that when the blacksmith wields the hammer, the hammer’s trajectory is more reproducible than the body configurations used to bring about that movement (Bernstein 1967). That original report had problems (e.g., the different repetitions of the movement were aligned for end-state rather than for initial body configuration) and presupposed that the variance of the hammer’s spatial position could be directly compared to the variance in body configuration. But the underlying notion bears out: Some combinations of degrees of freedom are more variable than others and such differences relate to the movement task. By embedding both the hammer’s position and the body configuration in joint space, the difference in variance between different combinations of degrees of freedom can be uncovered using the method of analysis of the uncontrolled manifold (UCM, Scholz and Schöner 1999). The empirical finding is that variance across repetitions of a movement is larger in those directions in joint space along which the hand position is invariant than in directions along which the hand position varies. Such a structuring of variance, the UCM signature of variance or “UCM effect”, has been observed in many different settings, for many different effector systems, and for a number of task variables in addition to the hand spatial position [reviewed in Latash et al. (2007), see also recent examples by Papi et al. (2015), de Vries et al. (2016), Greve et al. (2017), Tuitert et al. (2017)]. The UCM signature of variance is often preserved in clinical populations [e.g., Reisman and Scholz (2003)] and observed early in development (Golenia et al. 2018). Understanding the origins of the UCM signature of variance is therefore an important theoretical challenge.

So how does the DoF problem manifest itself at the level of the neural processes that generate movement and drive muscular activation? One idea is that movement plans are generated by populations of neurons in terms of spatial “task” variables like the hand’s movement direction (Georgopoulos et al. 1986; Schwartz 2007). The time course of population activation in motor cortex reflects, in that picture, the time course of the planned movement of the hand in space (Churchland et al. 2012). Theoretical models account neural processing at this task level through models of neural oscillation that generate a virtual trajectory of the hand in space (Martin et al. 2009; Rokni and Sompolinsky 2012). The hypothesis is that this time course at the task level is then transformed kinematically into a time course of motor commands to the muscles.

In a previous theoretical analysis (Martin et al. 2009), we examined some of the consequences of this hypothesis that the DoF problem is solved through a kinematic transformation. In the model, a virtual end-effector trajectory that connects an initial to a target position of the hand was generated through an oscillatory dynamics, which remained active for only a single cycle. The activation and deactivation of the oscillator were controlled by a competitive neural dynamics. The virtual trajectory of the end-effector was transformed into virtual trajectories of joint angles that specified motor commands for the muscles converging on each joint. That transformation included a kinematic pseudo-inverse, but also made use of couplings among the virtual joint variables that selectively stabilized those directions in joint space that impact on the end-effector position.

In the analysis, we examined the implications of the postulated kinematic solution to the DoF problem in terms of the mean joint trajectory. The model predicted, for instance, a relatively large amount of self-motion, that is, of motion in joint space that does not transport the hand in space. This prediction was confirmed by comparison with data from two experiments. We also compared different variants of the hypothesis. For instance, combining an inverse dynamics model that compensates for interaction torques with the same kinematic solution to the DoF problem led to the prediction of very little self-motion, too little to be experimentally valid.

We examined the effect of mechanical perturbations that induce motor-equivalent solutions to the DoF problem in which, following the perturbation, a new combination of joint angles is used to realize the same motor goal at the level of the hand in space. Motor equivalence, we showed, requires a form of “back-coupling” in which the motor command sent to the muscles is updated based on the actual, sensed joint configuration that emerges from the perturbed movement.

**Fig. 1:**
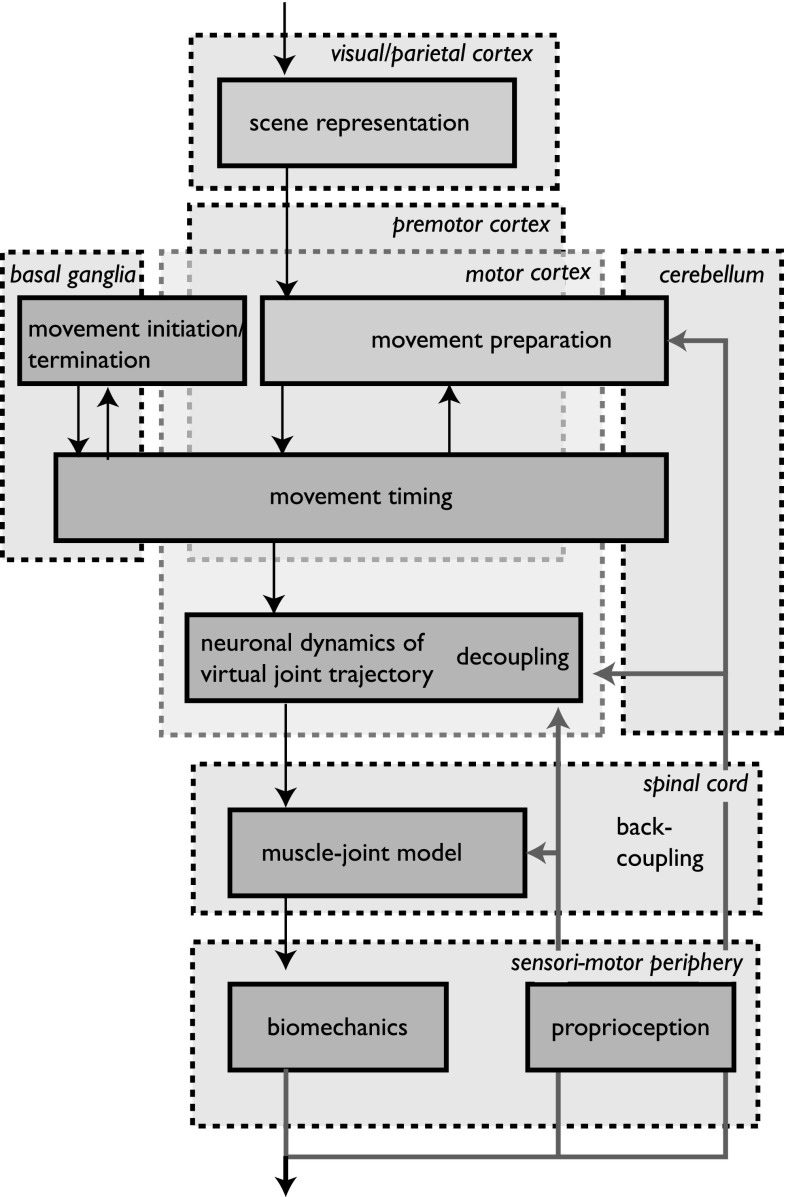
A survey over the model: Only the processes represented by blue boxes are modeled. The scene representation and movement preparation processes (gray boxes) are replaced by parameter values, but have been modeled elsewhere (Zibner et al. 2011, 2015). An approximate mapping onto structures of the CNS is sketched by the boxes in light gray. Different forms of recurrent loops are indicated by red arrows (color figure online)

**Fig. 2:**
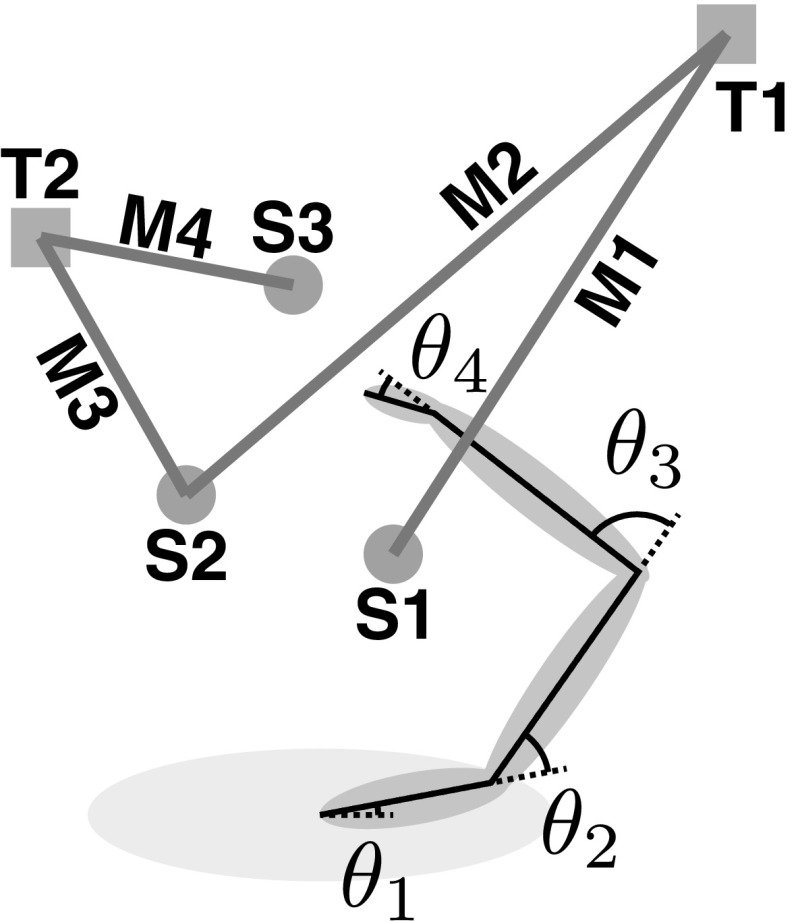
Experimental setup and kinematics

Random perturbations by neural or muscle noise were not addressed in this earlier work, however, which did not, therefore, establish an account for the UCM structure of variance. The present paper is aimed to do just that. Variability is commonly interpreted as a signature of the control stability of movement (Latash et al. 2007). The structure of variance in joint or muscle space is therefore thought to reflect the control priorities of the CNS. But is that conceptual understanding right? By injecting noise sources into different component processes of the multi-DoF movement generation model (Martin et al. 2009) and systematically examining their effect on the structure of variance in joint space, we provide an account for how the structure of control is reflected in the structure of variance.

## Model

Figure 1 provides a survey over the model, which has been published earlier (Martin et al. 2009). The model is formulated concretely for the planar four-degree-of-freedom arm controlling a two-dimensional end-effector that was probed experimentally (see Fig. 2). The model starts with a parametric description of movement plans that capture the outcome of *movement preparation*, which is formalized as a neural representation of the *direction* and *extent* of the hand’s movement in space. The processes through which a neural scene representation of the movement environment may be derived from visual information are described in other work (Zibner et al. 2011), as are the processes needed to extract movement parameters from such a scene representation (Erlhagen and Schöner 2002; Zibner et al. 2015). The initiation and termination of a discrete movement are modeled by a neural dynamic switching process (Schöner 1990; Richter et al. 2012). The three main parts of the model that relate directly to the degree of freedom problem and contribute to the variance structure of pointing movements are (1) the “timing” system that generates a timed virtual trajectory in end-effector space (Schöner 2002; Rokni and Sompolinsky 2012), (2) the “neural dynamics of the virtual joint trajectory” that transforms the virtual end-effector trajectory into joint space (Schöner 1994; Martin et al. 2009), and (3) the “muscle-joint model” that impacts on the biomechanical dynamics of the arm and captures spinal feedback loops (Gribble et al. 1998). Noise is injected into each of those modules as described below.

### Neural dynamics of movement initiation and termination

A movement is initiated by switching from the resting to the movement state and terminated by switching back. These two states are represented by dynamic neural nodes, $u_\mathrm {r}$ and $u_\mathrm {m}$, subject to the competitive neural dynamics

1 $$\begin{aligned} \tau _{\mathrm {m}} \dot{u}_{\mathrm {m}}= & {} -u_{\mathrm {m}} + h + I_{\mathrm {m}} - \sigma (u_{\mathrm {r}}) \nonumber \\ \tau _{\mathrm {r}} \dot{u}_{\mathrm {r}}= & {} -u_{\mathrm {r}} + h + I_{\mathrm {r}} - \sigma (u_{\mathrm {m}}) \end{aligned}$$

with negative resting level, *h*, and sigmoidal nonlinearity, $\sigma (u)=1/(1+\exp [- 100\ u])$. The input, $I_{\mathrm {m}}$, is an external “go” signal. The input, $I_{\mathrm {r}}$, depends on the predicted state of the end-effector such that it reactivates the resting state at the end of the movement (see Martin et al. 2009, for details).

### Timing dynamics

A movement is generated through a virtual velocity profile, represented in end-effector space by the two-dimensional virtual end-effector velocity vector, $\mathbf {u} = (u_1, u_2)$, and modeled as a stable limit cycle of a Hopf normal form oscillator that is topologically equivalent to a wide class of oscillating dynamical systems (Perko 2001):

2 $$\begin{aligned} f_\mathrm {h}(u_i, z_i)&= \begin{pmatrix} \alpha _\mathrm {h} &{} - \omega _\mathrm {h} \\ \omega _\mathrm {h} &{} \alpha _\mathrm {h} \end{pmatrix} \begin{pmatrix} u_i - U_i \\ z_i \end{pmatrix} \nonumber \\&\quad - \alpha _\mathrm {h} U^2_i \left( (u_i - U_i)^2 + z_i^2 \right) \begin{pmatrix} u_i - U_i \\ z_i \end{pmatrix}, \end{aligned}$$

where $z_i$ is a second, auxiliary dynamical variable, and $i=1,2$ spans the two dimensions of end-effector space. The parameter, $\omega _\mathrm {h}$, sets the frequency of the limit cycle oscillation, $\alpha _\mathrm {h}$ its relaxation rate, and $U_i$ its amplitude. Note that this limit cycle dynamics is only active while $u_\mathrm {m}$ is above threshold, typically for a single cycle. When the $u_\mathrm {r}$ is active, a fixed point attractor at $\mathbf {u}=0$ arises instead. The complete timing dynamics is given by

3 $$\begin{aligned} \begin{pmatrix} \dot{u}_i \\ \dot{z}_i \end{pmatrix} = \sigma (u_\mathrm {m}) f_\mathrm {h}(u, z) + \sigma (u_\mathrm {r}) \begin{pmatrix} - u_i \\ - z_i \end{pmatrix} + \psi _o, \end{aligned}$$

where $\psi _o$ is time-correlated noise (see Sect. 2.5). This noise term generates inhomogeneous variations of the time course of the movement that induce variations of total movement time, but go beyond a mere rescaling of the entire time course.

### Neural dynamics of the virtual joint trajectory

The virtual end-effector velocity, $\mathbf {u}$, is low-pass filtered to provide a signal, $\dot{\mathbf v}$, for the virtual end-effector acceleration

4 $$\begin{aligned} \dot{\mathbf v} = -\alpha \left( {\mathbf v } - {\mathbf u} \right) \end{aligned}$$

that is transformed into a virtual joint acceleration vector,

5 $$\begin{aligned} \ddot{{\lambda }} \sim \mathbf {J}^+({\lambda }) \left( \dot{\mathbf {v}} - \dot{\mathbf {J}}({\lambda }) \dot{{\lambda }} \right) \end{aligned}$$

where $\mathbf {J}^+$ indicates the Moore–Penrose pseudo-inverse of the manipulator Jacobian matrix, $\mathbf {J}({\theta }) = \partial {\mathbf {p}} / \partial {{\theta }}$, that relates changes in joint configuration, ${\theta }$, to changes in end-effector position, $\mathbf {p}$.

As a second-order dynamical system, this equation attracts the virtual joint velocity, $\dot{{\lambda }}$, toward the direction in which it produces the virtual end-effector velocity, $\mathbf {v}$. Orthogonal to this direction, the vector field of the dynamics is zero. In this orthogonal direction, we add a contribution that couples the real, time-delayed joint configuration, $\theta _\mathrm {d}$, back into the virtual configuration

6 $$\begin{aligned} \ddot{\lambda }\sim \mathbf E \left( - \beta _{n_1} \mathbf E^T (\lambda - \theta _d) - \beta _{n_2} \mathbf E^T (\dot{\lambda }- \dot{\theta }_d) - \dot{\mathbf E}^T \dot{\lambda }\right) , \end{aligned}$$

where $\beta$ are gain factors and $\mathbf E(\lambda ) \in \mathbb R^{4 \times 2}$ is the projection matrix onto the orthogonal complement of the null space of $\mathbf J(\lambda )$. This “back-coupling” makes that the virtual joint vector, $\lambda$, yields to the real joint vector, $\theta$, within the null space of the end-effector position (see Martin et al. 2009, for details).

The dynamics of the virtual joint velocity

7 $$\begin{aligned} \ddot{\lambda }&= \mathbf {J}^+ \left( \dot{\mathbf {v}} - \dot{\mathbf {J}} \dot{{\lambda }} \right) \nonumber \\&\quad + \mathbf E \left( - \beta _{n_1} \mathbf E^T (\lambda - \theta _d) - \beta _{n_2} \mathbf E^T (\dot{\lambda }{-} \dot{\theta }_d) - \dot{\mathbf E}^T \dot{\lambda }\right) {+} \psi _\lambda \end{aligned}$$

consists, therefore, of two orthogonal contributions, the first lying in the subspace in which the virtual joint velocity moves the end-effector (“task-relevant”), the second lying in the subspace in which the virtual joint velocity does not move the end-effector (“task-irrelevant”). $\psi _\lambda$ is “neuronal noise” (see Sect. 2.5).

### Muscle and biomechanical dynamics

The virtual joint vector, $\lambda (t)$, represents the descending motor commands that modulate muscle activation in the spinal cord. Muscle force generation is modeled based on Gribble et al. (1998), simplified by neglecting the dynamics of force buildup (“calcium dynamics”) and the delays in the stretch reflex. The torque generated by four virtual muscle groups

8 $$\begin{aligned} T_i&= K_\mathrm {l} \cdot \Bigl ( \exp ([K_\mathrm {nl} \cdot (\theta _{i}-\lambda _{i}^{p})]^{+}-1) \nonumber \\&\quad - \exp ([K_\mathrm {nl} \cdot (-\theta _{i}+\lambda _{i}^{m})]^{+}-1) \Bigr ) \nonumber \\&\quad + \mu _\mathrm {bl} \cdot \mathrm {asinh}(\dot{\theta }_{i}-\dot{\lambda }_{i}) + \mu _\mathrm {rn} \cdot \dot{\theta }_{i} \end{aligned}$$

contains contribution of agonist muscles, $\lambda _{i}^{p}=\lambda _{i}-Co$, and antagonist muscles, $\lambda _{i}^{m}=\lambda _{i}+Co$, whose motor commands are offset by a constant co-contraction command, *Co*. The semi-linear threshold function is signified by $[... ]^+$. Joint angles are designated as $\theta _i$, and joint velocities as $\dot{\theta }_i$. There are both linear, $K_\mathrm {l}$, and nonlinear stiffness parameters, $K_\mathrm {nl}$, as well as two types of viscosity, $\mu _\mathrm {bl,rl}$. See the appendices in Martin et al. (2009) for detailed arguments for the various approximations.

The effects of multi-articulatory muscles are emulated by including off-diagonal elements in the impedance matrix, $\mathbf {Z}$, that distributes muscle torques $\mathbf {T}=(T_1, \dots , T_4)$ onto active joint torques, $\mathbf {T}_\mathrm {m}$:

9 $$\begin{aligned} \mathbf {T}_\mathrm {m} = \mathbf {Z } \cdot \mathbf {T} + \psi _m, \end{aligned}$$

where $\psi _m$ is a vector of time-correlated motor noise (see Sect. 2.5).

The biomechanics of the arm moving in the horizontal plane are modeled with four degrees of freedom, representing rotational motion of the sternoclavicular, shoulder, elbow, and wrist joints. The forward kinematic mapping relating joint movements to movements of the pointer was derived using the screw theory framework (Murray et al. 1994). The equation of motion

10 $$\begin{aligned} \mathbf {M} \ddot{\theta }+ \mathbf {C} \dot{\theta }= \mathbf {T}_\mathrm {m} \end{aligned}$$

relating active joint torques, $\mathbf {T}_\mathrm {m}$, to joint accelerations, $\ddot{\theta }$, was derived following the Lagrangian approach.

### Noise

Movement is variable. When the same movement task is performed repeatedly, the variability across trials reflects the balance between stabilization mechanisms and sources of noise. It is generally assumed that different sources of noise contribute (Schöner and Kelso 1988). Force generation by muscles is noisy and has been shown to be a major factor in movement variability (van Beers et al. 2004). The neural processes driving movement generation are prone to random fluctuations characteristic of all neural activity (Stein et al. 2005).

To examine the impact of noise on movement variability, we model noise sources at three levels. (1) “Timing noise”: The generation of the timing signal at the end-effector is subjected to noise (see Eq. 3). (2) “Neural noise”: The transformation to a virtual joint trajectory is subjected to noise (see Eq. 7). (3) “Muscle noise”: Muscle force generation is assumed noisy (see Eq. 9). Noise is modeled as additive, time-correlated (Ornstein-Uhlenbeck) stochastic contributions, $\psi (t)$, to the respective dynamics:

11 $$\begin{aligned} \tau _{\psi } \cdot \dot{\psi }=-\psi +n_{\psi } \cdot \zeta , \end{aligned}$$

where $\zeta$ is Gaussian white noise, $\tau _{\psi }$ the correlation time, and $n_{\psi }$ noise strength. Noise at the higher levels of movement planning was considered, but found to have minimal influence on the trial-to-trial variability of the executed movement trajectory and was thus dropped from consideration. Because our model generates relatively fast and thus largely ballistic movements, sensory noise and online updating were neglected.

A fourth source of variance is the variation in the initial end-effector position and joint configuration across trials that is observed in the experiment. The three sources of noise listed above do not account for that variance. We modeled variance of the initial joint configuration by adding a random vector drawn from a uniform distribution to the initial virtual joint configuration and then letting the system relax to the fixed point of system while the timing signal was at rest (Eq. 3).

### Parameter values

The only parameters adjusted to fit data in this paper are the strengths of the noise sources. These were adjusted by hand to achieve appropriate orders of magnitude of the resultant variance. In some simulations, noise sources were selectively set to zero to demonstrate their role.

All other model parameter values were taken from Martin et al. (2009, listed in the appendices of that paper). We use the “reference parameter set” that was found in that paper to account for a large set of experimental signatures. We did not adjust these parameter values to account for the variance data. In some simulations, we set particular terms to zero to probe their effect. This is explained for each simulation.

## Experimental and simulation methods

We obtained a set of movement data from three participants to compare the model to experiment. Our goal was qualitative comparison to assess which features of the data were robust and reproducible and could thus be meaningfully expected to be matched by the model. Fitting the individual datasets is not consistent with the strategy for choice of parameter values (see above).

### Experimental protocol

Experiments on human movement were performed by one of the authors (VM) at the laboratory of our late colleague Dr. John Scholz at the University of Delaware. Three volunteers between 21 and 35 years of age volunteered to participate in this study. Participants gave informed consent. All three participants were right-handed and used their right arm to reach the targets.

Participants sat on chair with a high backrest. Trunk movements were restrained by a chest harness that was firmly attached to the backrest of the chair. Subjects wore a hand brace containing a stylus that was aligned with the extended index finger. The table height was adjusted so that the right arm rested on it horizontally when in the starting position.

Participants performed reaching movements from three different starting locations to two different targets (see Fig. 2). Targets T1 and T2 were positioned at 90% arm length along two lines passing through the right acromion process at angles of $\pm \, 40^\circ$ to the side. Starting locations S1 and S2 were 7.8 cm anterior from the sternum and the right acromion, and starting location S3 was at 50% arm length along a line passing through the right acromion, rotated $20^\circ$ rightward. The initial arm configuration was marked for each movement, and subjects were asked to replicate this configuration as closely as possible between repetitions. Four different combinations of starting and target locations were used: M1: S1 $\rightarrow$ T1, M2: S2 $\rightarrow$ T1, M3: S2 $\rightarrow$ T2, and M4: S3 $\rightarrow$ T2. Subjects performed 25 repetitions in each condition in randomized order.

Participants were asked to reach for the target with the tip of the stylus as accurately as possible, but in one continuous movement without pausing or correcting for misses. The movement time was determined during test trials in which participants were instructed to move as quickly as possible. During the actual experiment, participants were asked to maintain this movement time, which was on the order of 500 ms for all participants. Movement time was measured and trials were repeated if the movement time deviated by more than 5% from the target movement time. Participants were able to practice movements under these instructions prior to data collection with verbal feedback from the experimenter on spatial and temporal accuracy.

Five reflective markers of 1.5 cm diameter were placed on the suprasternal notch, just below the lateral tip of the acromion, the lateral epicondyle of the humerus, the radial styloid process of the wrist and the tip of the handheld stylus. Additional markers were placed at the center of the each target. Kinematic data from these markers were recorded using a VICON-370 motion capture system at a sampling rate of 120 Hz. The data were filtered using a two-directional, fourth-order butterworth low-pass filter with a cutoff frequency of 5 Hz.

### Simulations

To simulate the model, the stochastic differential equations were solved numerically in MATLAB (MathWorks, Inc), using the stochastic Euler method with a time step of 2 ms (Kloeden and Platen 1999). The trajectories obtained from repeated numerical simulation of the model were analyzed using the same procedures as those applied to experimental data.

### Time normalization

To estimate variance of movement trajectories across repetitions, trajectories were normalized in time between movement initiation and termination. Such time normalization uses a homogeneous rescaling of the time course of all movement trajectories to eliminate variation in movement time. To normalize the time, we first calculated the end-effector velocity by numerical differentiation. For each repetition, movement initiation was defined as the point in time at which the end-effector velocity first reached a threshold of 1% of its peak value. Movement termination was defined as the time when $\mathbf v$ dropped below 3% of its peak value. The time-normalized trajectories were resampled to 100 data points using spline interpolation.

The estimates of movement onset and termination are a first possible source of error, which may have different effects on estimates of variance at different moments in time. In particular, the same misalignment in time has a larger effect on the variance of end-effector position at larger movement speeds. Therefore, position errors along the main movement axis will be larger near peak velocity in the middle of the movement and smaller at low speeds early and late in a movement. Timing-induced position errors orthogonal to the main movement axis, on the other hand, depend upon the curvature of the path, which tends to be bimodal. We refer to these different axes *movement extent*, defined as “end-effector position along the line from the start to the target position”, and *movement direction*, defined as “end-effector position orthogonal to the line from the start to the target position.”

**Fig. 3:**
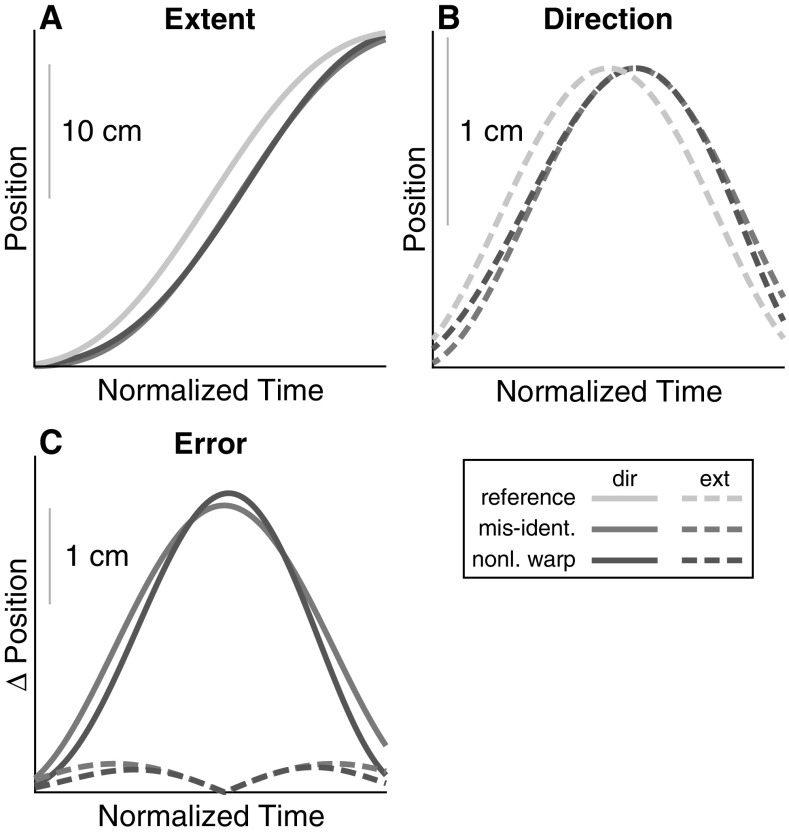
Effect of temporal misalignment (blue) and deviation from homogeneous rescaling in time (purple) on a reference end-effector trajectory (green). Panel A shows movement extent, i.e., movement along the axis between the start and end positions. Panel B shows movement direction, i.e., movement orthogonal to extent. Panel C shows the errors (deviations from reference trajectory) induced by such timing issues (color figure online)

The effect of time normalization errors is illustrated schematically in Fig. 3, comparing a reference end-effector trajectory (green line in Panel A and B) with a copy of itself shifted in time to model misidentification of movement onset and termination (blue line in Panel A and B). The error induced by time normalization, i.e., the difference between the two trajectories, is bell-shaped for movement extent (solid blue line in Panel C) and bimodal for movement direction (dashed blue line in Panel C). A deviation from the assumption of homogeneous rescaling leads to similar errors, as shown by the red lines in Panel C. Here, time was warped nonlinearly by adding an quadratic error term that leaves movement onset and termination times invariant but warping the time in between. This characteristic time dependence of the errors induced by the two forms of timing misalignment is relevant when examining time courses of variance.

### Structure of variance in joint space

To analyze the structure of variance within the four-dimensional joint space, we used two methods of analysis, the method of the uncontrolled manifold (UCM) and a method based on principle component analysis (PCA).

*UCM* The UCM method compares variance in task-relevant and task-irrelevant directions in joint space. Originally introduced in Scholz and Schöner (1999), mathematically refined formulations of the UCM method of analysis of variance have been published (Schöner and Scholz 2007; Yen and Chang 2010; Verrel 2011; Campolo et al. 2013), so that only a brief sketch need be provided here. For any potential task variable $\phi =f(\theta )$, let $J_\phi = \frac{\partial \phi }{\partial \theta }$ be the Jacobian matrix that represents locally how the task variable, $\phi$, depends on joint angles, $\theta$. Let $E_\Vert$ and $E_\perp$ be the orthogonal projection matrices onto the null space of $J_\phi$ and its orthogonal complement. Given a sample covariance matrix $\widehat{\varSigma }$, the variance along and orthogonal to the UCM is estimated by the traces

12 $$\begin{aligned} V_\Vert = \frac{1}{k_\Vert } \mathrm {tr} \left( E^T_\Vert \widehat{\varSigma } E_\Vert \right) \quad \mathrm {and} \quad V_\perp = \frac{1}{k_\perp } \mathrm {tr} \left( E^T_\perp \widehat{\varSigma } E_\perp \right) , \end{aligned}$$

where $k_\Vert$ and $k_\perp$ are the dimensions of the task-irrelevant and task-relevant subspaces. Task variables can be examined separately. In particular, we examine the task variables *movement extent* and *movement direction*. For each movement condition and at each point of normalized time, we calculate the sample covariance matrix around the mean and then the UCM measures given in Eq. 12.

*PCA* While the UCM method of analysis tests particular hypotheses about task variables that structure variance, an alternative is to first estimate the structure of variance in a data-driven way (e.g., using principle component analysis, PCA) and then interpret the observed structure relative to different hypotheses.^1^ In many studies, there are not enough data to perform such a data-driven search for the structure of variance. The simple nature of the present experiment enables us to obtain 25 repetitions of each movement, so that data-driven analysis is feasible. In the model, the number of repetitions is unlimited, of course. We can use the data-driven approach to observe how the structure of variance depends on different components of the model (see the next section). Providing a second, convergent method of analysis to characterize the structure of variance in joint space can serve to consolidate the empirical and theoretical results.

We performed such an analysis for the endpoint of the movement. The covariance matrix in joint space was constructed across movement repetitions, and a PCA was performed (Daffertshofer et al. 2004). This yields an ordered set of basis vectors of joint space in which each basis vector captures the maximal amount of remaining variance.

To test the hypothesis that variance is compressed in directions in which the task variable varies, we examined the degree of alignment of basis vectors with the task manifold by computing the angles between the two-dimensional task manifold and each of the two main principal components.

**Fig. 4:**
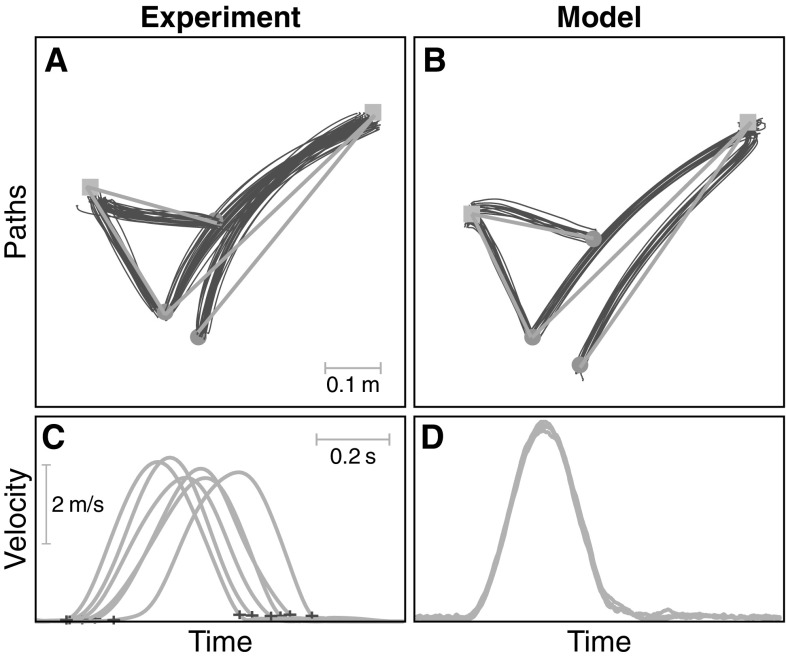
Top: end-effector paths from different trials of the four movements for a typical participant (left) and multiple simulation runs of the model (right). Sample profiles are shown on bottom, unnormalized for the participant (left), while movement time does not vary for the model (right)

## Results

### Variance of end-effector trajectories and role of alignment errors

Figure 4 illustrates the paths of the end-effector for a typical participant and for the model simulations. The experimental movement paths are slightly curved, depending on movement direction. Overall, these features are well reproduced by the model, although the curvature for movement 4 (see Fig. 2) is in the wrong direction. The velocity profiles show the characteristic bell shape of pointing movements (Morasso 1981; Flash and Hogan 1985). The experimental velocity profiles exhibit high variability in onset and termination, highlighting the need for time normalization. Movement time in the model is not varied.

**Fig. 5:**
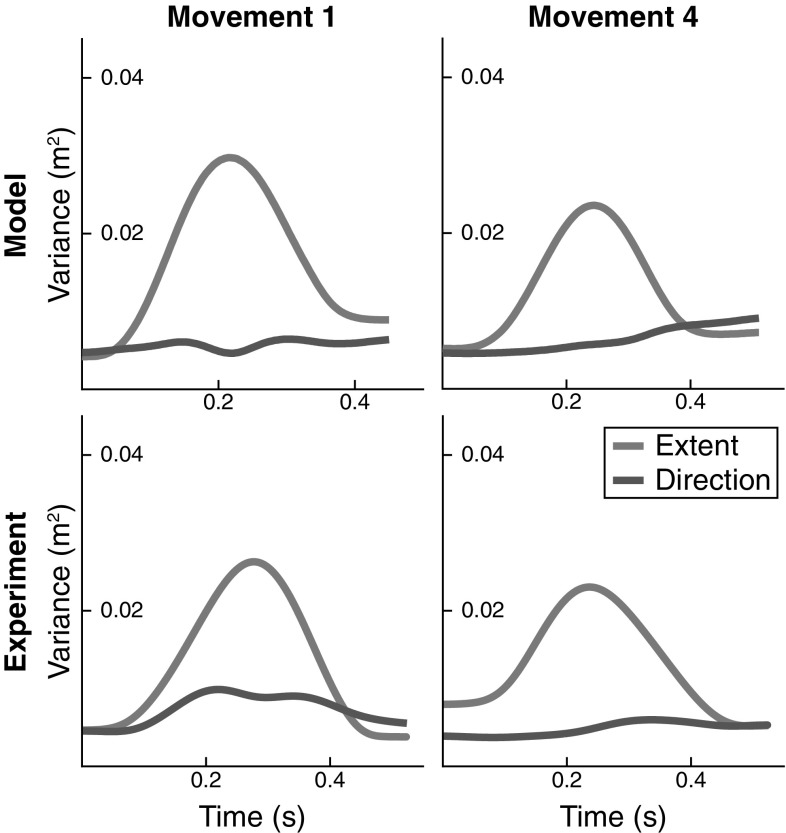
End-effector variance for the model (top) and experiment (bottom) separately for the extent (blue) and direction (red) components (color figure online)

The variance of the end-effector trajectories is illustrated in Fig. 5. To compute variance across repetitions, time was normalized. End-effector variance is broken down into variance in movement direction and movement extent for two movements (1 and 3) and one participant. Variance of movement extent is strongly bell-shaped with a peak near the peak velocity of the movement. Variance of movement direction is generally much smaller than variance of movement extent. Variance obtained from the model matches the experimentally observed pattern.

**Fig. 6:**
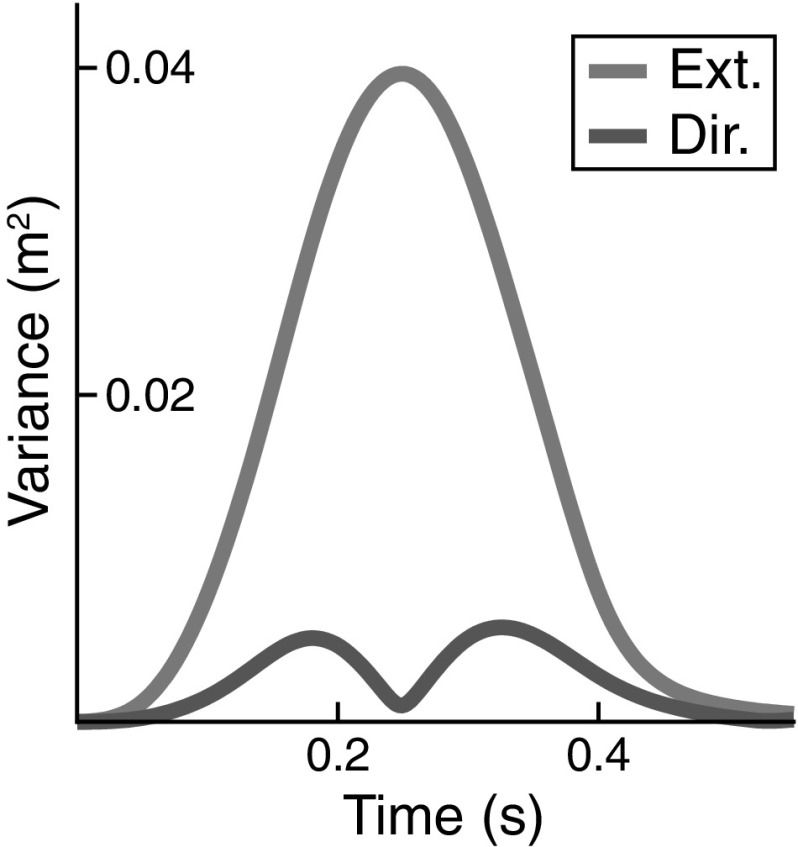
Illustration of the effect of trial misalignment on end-effector variance in the model for movement 1

To study how timing variability and misalignment impact on end-effector variability, we ran two kinds of simulations. In one, we added uniformly distributed random noise to the movement initiation and termination times. This emulated misalignment of different trials. Figure 6 shows the end-effector variance from the trial misalignment simulation study, where random perturbations of movement initiation and termination were the only source of variability, separated into movement direction and extent. As expected based on the results from the simple example in Fig. 3, the movement extent variability induced by trial misalignment is strongly bell-shaped. For the movement direction hypothesis, the effect is much smaller, with a bimodal shape containing a significant dip around peak velocity. At movement initiation and termination, there is almost no variability, as expected in the absence of other noise sources. Together, the simulations in Figs. 6 and 5 indicate that alignment errors contribute to end-effector variance, but are not the only, or even a necessary element capable of explaining the structure of end-effector variance.

**Fig. 7:**
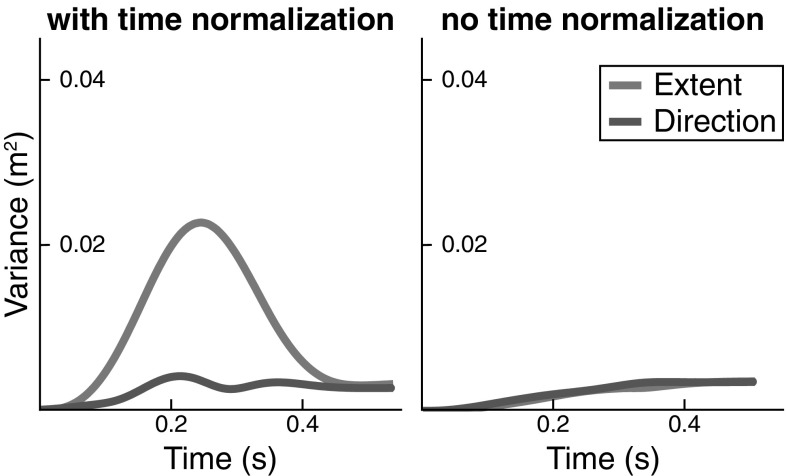
End-effector variance obtained from simulation when a strong timing error is the only noise source in the model (Movement 6)

In the second class of simulations, we induced variance of timing that was inhomogeneous in time by strongly increasing the strength of the timing noise, $\psi _o$ (from 0.008 to 0.05, see Eq. 3). All other noise sources were set to zero. Figure 7 shows the effect on end-effector variance of inhomogeneous timing variance. With time normalization (left), the bell-shaped modulation of end-effector variance in movement extent is observed, while movement direction shows a smaller effect. The variance of the end-effector after the movement ends does not return to zero, making the bell shape asymmetrical. This is due to the fundamental lack of stability of the timer (oscillator) along its phase, so that drift occurs over time. When time is not normalized (right panel of Fig. 7), that monotonic increase of variance due to phase drift is clearly visible. Note that now there is no bell shape of variance in either component, clearly pointing to the origin of that shape in sources of noise (misalignment and neural/muscle noise) other than timing noise itself.

Clearly, time normalization is partly responsible for the strong modulation of end-effector variance with time, inducing a bell-shaped profile, especially for the extent component, that reflects the direction in end-effector space along which movement actually occurs. There is not way, however, that time normalization can be avoided, however, as we need to compare matching moments in time across trials and that entails aligning trials by on- and offset. Fortunately, the model is able to capture the modulation of variance even when misalignment is neglected.

### Variance in joint trajectories

**Fig. 8:**
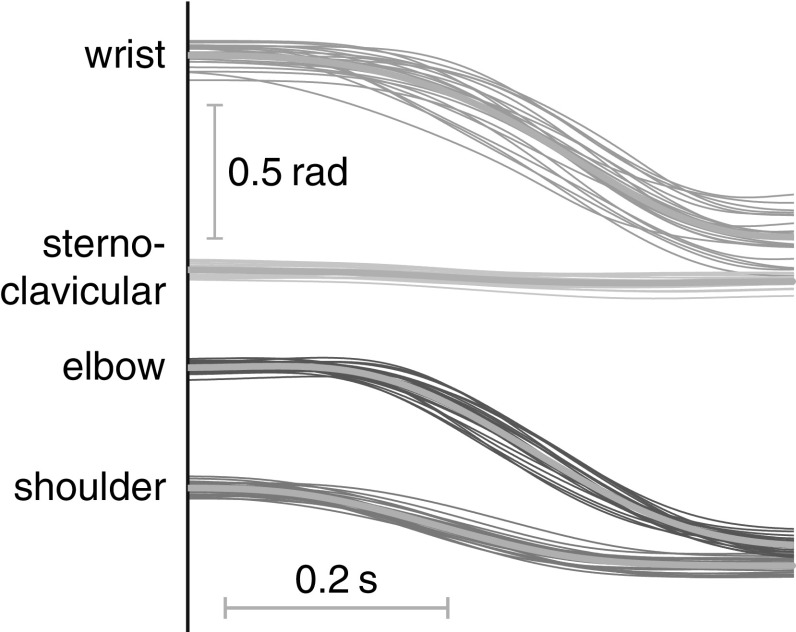
Trajectories of the four joint angles across trials for one movement of one participant

Figure 8 overlays the joint angle trajectories of all trials for one exemplary participant and movement. Variability of shoulder and elbow joint trajectories appears to be larger in mid-movement, consistent with a somewhat bell-shaped profile of joint trajectory variance. The trajectory of the sternoclavicular joint is close to flat and has little variance. Variability of the wrist joint trajectory is large overall and visibly larger near the end of the movement than at the beginning.

**Fig. 9:**
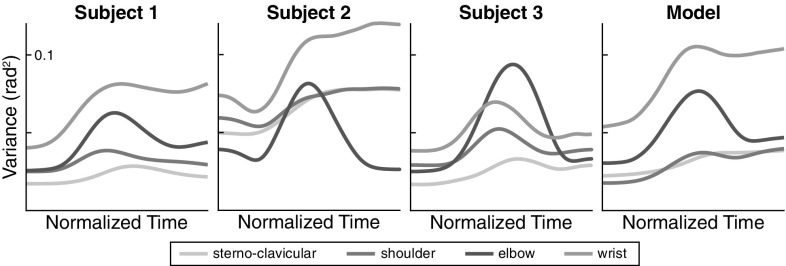
Joint angle variance computed across trials as a function of normalized time for movement 4 for each participant and for the model. Joint angles are color-coded as in Fig. 8

Average joint angle variance of movement 4 is plotted across normalized time in Fig. 9 for all three participants and for the model simulations. This figure illustrates differences in joint trajectory variability across participants. Some features emerge as common traits. Many joint variance profiles are roughly bell-shaped, with a distinctive peak of variance in mid-movement. This is similar to the temporal profiles of end-effector variance (for movement extent, see Fig. 5), suggesting that some of the mid-movement variability might be due to time normalization as discussed previously. A distinct feature is that for most joints, variance increases over time and remains larger at the end of the movement than at the beginning. This stands in contrast to the variance of end-effector trajectories, in which no such increase was observed (see Fig. 5). This suggest that joint angle variance increases in those directions of joint space that does not affect the end-effector variance, i.e., along the task-equivalent manifold, but not in other directions of joint space. Note that the model captures these features that are robust across participants.

### UCM analysis of variance

**Fig. 10:**
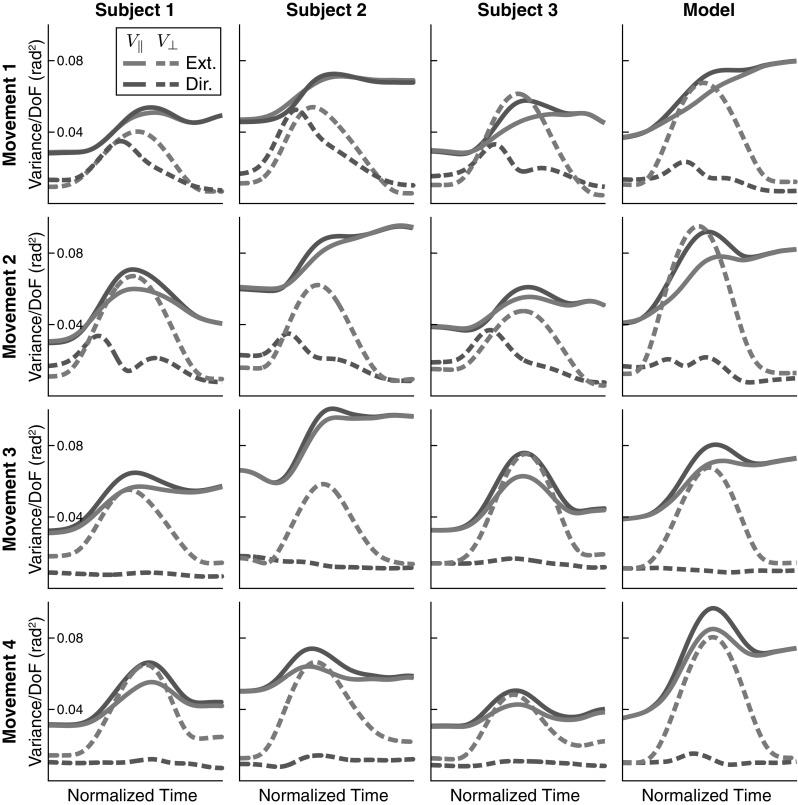
UCM analysis of variance for all three participants (columns 1–3) and for the model (column 4), as well as for all four movements (rows). In each case, the solid lines are variance parallel to the UCM and the dashed lines are variance orthogonal to the UCM. The UCM is computed either relative to the task variable *movement extent* (blue lines) or to the task variable *movement direction* (purple lines) (color figure online)

To formalize the intuition that the variance of joint angle trajectories conserves the end-effector trajectory, we employ the UCM method of analysis. The results are shown in Fig. 10 for all four movements, all participants, and the model. The participants differ in the absolute levels of the different components of variance, but three main features that are invariant across participants and movements emerge. First, $V_\perp$ at movement termination is similar or slightly smaller than $V_\perp$ movement onset. This component of variance has a peak in mid-movement, which is of medium size for the task variable *movement direction* and pronounced for the task variable *movement extent*. Second, $V_{\Vert }$ is substantially higher at movement termination than at movement onset, with a medium-sized peak in mid-movement. Third, $V_{\Vert }$ is generally larger than $V_\perp$, with the exception of subject 3 in mid-movement for the movement extent variable, where $V_\perp$ temporarily exceeds $V_{\Vert }$. Because movement extent captures the direction along which the hand is moved, the peak of variance in $V_\perp$ reflects the peak in variance of the hand’s position along its path, indicating that this increase in variance is largely due to time normalization (see Fig. 7).

These three major characteristics are reproduced very well by the model. Note that other features are not consistent across movements and participants. For instance, the orthogonal variance of the task variable direction is more strongly modulated in time for movements 1 and 2 than for movements 3 and 4 and that modulation is not well captured by the model. The stronger modulation is due to the more curved movement paths of movements 1 and 2 (Fig. 4), which create stronger modulation of movement direction, and—through misalignment—an increase of orthogonal variance of direction in the middle of the movement. The model does not reproduce the curved paths so well, perhaps because control is too stiff in the model overall.

**Fig. 11:**
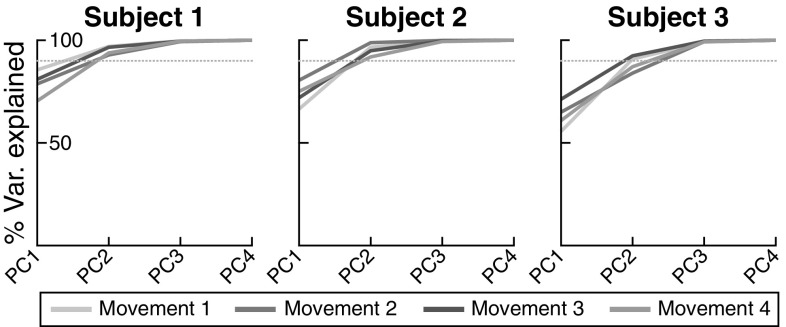
Proportion of variance in joint space at movement termination explained by consecutively adding principles components across all three participants and each movement (color-coded). The dotted line indicates a 90% level as a visual guide (color figure online)

### PCA approach to discovery of structure of variance

To probe inter-joint coordination further, we employ PCA of the joint angle variance at the movement termination. Figure 11 shows for each participant, and each movement, the proportion of total variance that is explained by consecutively adding principle components (PCs). The first two components explain most of the variance in all cases.

The angles between each PC and the UCM for the task variable end-effector position in space are listed in Table 1. Consistent with the UCM analysis, these angles are near $0^\circ$ for the first two PCs and near $90^\circ$ for the remaining two PCs. So the PCA approach to discovery of the structure of variance is in close agreement with the UCM hypothesis testing approach. Below, we will use this analysis to study the causes of the structure of variance in the model.

**Table 1:** Angles between the four principal components obtained at movement termination and the UCM for the task variable end-effector position

Movement	PC1	PC2	PC3	PC4
1	2.4	0.8	87.8	88.5
2	2.4	4.4	82.2	87.3
3	1.4	3.8	86.8	87.6
4	4.9	3.3	84.1	89.1

### What causes the UCM effect?

The model enables us to identify possible causes of the UCM structure of variance by varying model components and documenting their influence on the structure of variance.

**Fig. 12:**
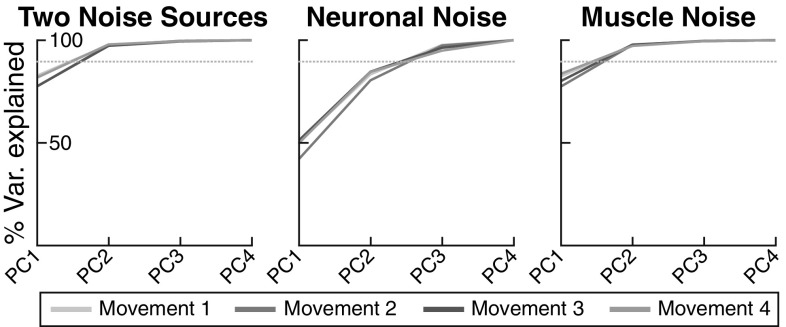
Proportion of variance in joint space at movement termination explained by consecutively adding principle components across for each movement (color-coded) for the model. Three variants of the model are compared. The dotted line indicates a 90% level as a visual guide (color figure online)

*Role of noise sources* Figure 12 shows the results of applying the PCA approach to data from model simulations. Model simulation results obtained from the reference parameter set (left panel) are generally similar to the experimental data (Fig. 11). When “neuronal noise” in the transformation of the timing signal to the virtual joint trajectory (see Sect. 2.5) is the only source of variance (middle panel), then the variance generated by the model is not highly structured. The first principal component explains only $\approx$ 50% of the total variance, substantially less than in the experimental data for subjects 1 and 2. When “muscle noise” is the only source of variance (right panel), the structure of variance is closer to the experimentally observed pattern for these two subjects. For subject 3, the first PC explains less variance compared to subjects 1 and 2, though still more than for the model without neuronal noise. It seems likely that a combination of the two noise sources, with reduced neuronal noise strength compared to the reference parameter set, would fit data from this subject better, but explaining inter-subject variability is beyond the scope of this study. The “timing noise” has a very weak effect on joint variance, so that its influence on the structure of variance is difficult to establish.

*Persistence of initial variance* One striking feature of the UCM analysis is that even at the beginning of the movement, there is already some structure of variance consistent with the UCM of the end-effector position (Fig. 10). This structure of variance reflects how the arm is initially configured by participants to place the hand at the desired starting location. In the model, we can study to which extent this initial structure of variance is preserved throughout the movement and contributes to the structure of variance over time.

**Fig. 13:**
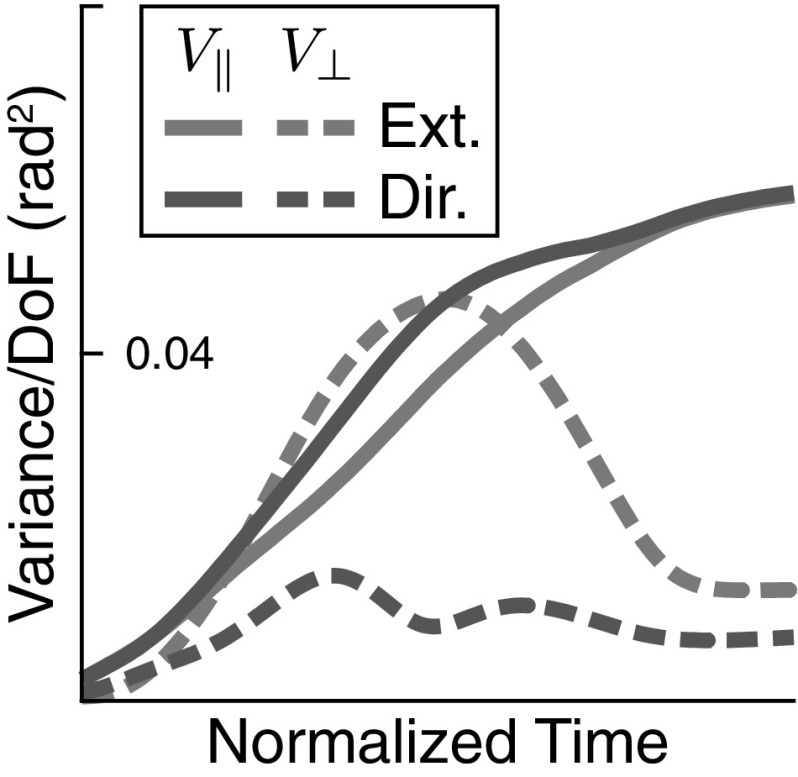
UCM structure of variance across time for a simulation of movement 1 in which the initial variance of end-effector and joint configuration has been removed (solid line: $V_{\Vert }$, dashed line: $V_\perp$) compared to the top right simulation in Fig. 10

In the model, the initial variance of end-effector and joint configuration was modeled by adding a noise term to the initial configuration and then simulating the model in the resting state for a while (see Sect. 2.5). When we remove this mechanism from the simulations, initial variance is indeed zero as seen in Fig. 13. By comparing this simulation with the corresponding simulation that includes variance of the initial configuration (movement 1, top right in Fig. 10), we make the following observations. First, the UCM structure of variance still emerges in the absence of initial variance. So the UCM structure is not caused by the initial structure of variance alone. Second, for the UCM component, initial variance adds variance throughout the movement. At the end of the movement, UCM variance for both task variables is 0.078 rad$^2$*with* and 0.058 rad$^2$*without* initial variance, a difference of 0.020 rad$^2$. The initial UCM variance is 0.036 rad$^2$, so some of the initial UCM variance is preserved across the movement. Third, the components of variance orthogonal to UCM are increased by initial variance early during the movement, but that increase does not persist. Thus, at the end of the movement, orthogonal variance for movement extent (red dashed) is 0.0073 rad$^2$ both *with* and *without* initial variance. For orthogonal variance of movement direction (blue dashed), it is 0.012 rad$^2$ in both conditions.

**Fig. 14:**
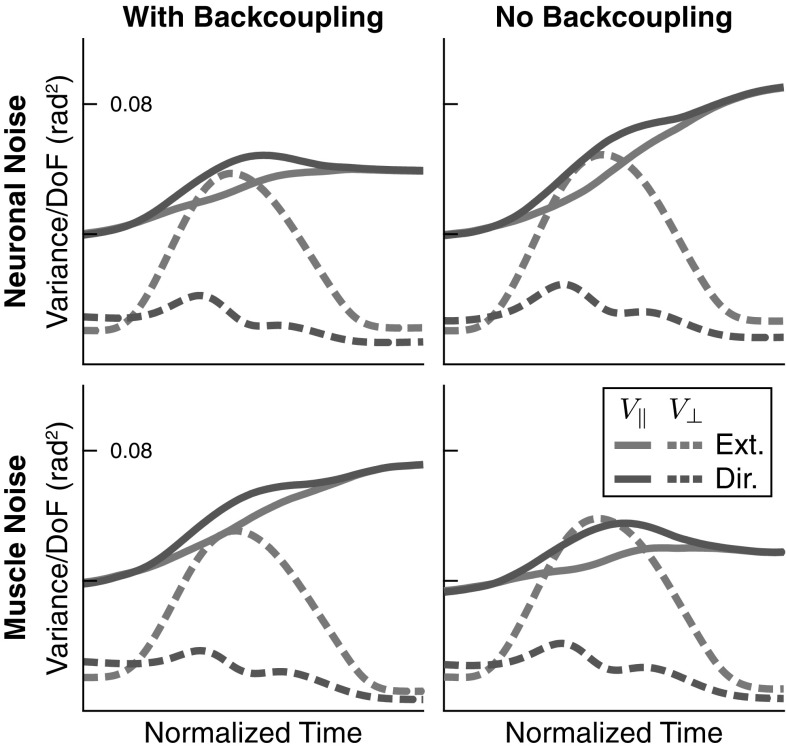
UCM variance obtained from the model when back-coupling is removed (right column) when the sole noise source is “neural noise” (top) or “muscle noise” (bottom). (Solid lines for $V_{\Vert }$, dashed lines for $V_\perp$, blue for extent, red for direction hypothesis) (color figure online)

*Role of back-coupling* Back-coupling is responsible for updating the virtual joint trajectory to yield to the real joint trajectory within the UCM. This mechanism was introduced to account for the emergence of motor-equivalent joint configuration in response to external perturbations (Martin et al. 2009). Noise is a source of perturbation, so we must establish how back-coupling affects the structure of variance in otherwise unperturbed movements. To do that, we compare the results of the model with back-coupling turned on and off: Fig. 14 contrasts simulations of the regular model with back-coupling (left column) with simulations in which the back-coupling terms in the model were set to zero ($\beta = 0$ in Eq. 6). Because back-coupling might interact differently with neuronal noise and motor noise, the comparison is made separately when only “neural noise” or only “muscle noise” is applied to the model.

In the task-relevant subspaces, variance remains more or less unaffected by both back-coupling and noise source and retains its characteristic shape. In the task-irrelevant subspaces, back-coupling interacts differently with neural than with muscle noise. When neuronal noise is the main source of variability (top row), back-coupling limits the growth of UMC variance toward the end of the movement. When muscle noise is the main source of variability (bottom row), back-coupling has the opposite effect of increasing UCM variance toward the end of the movement.

**Fig. 15:**
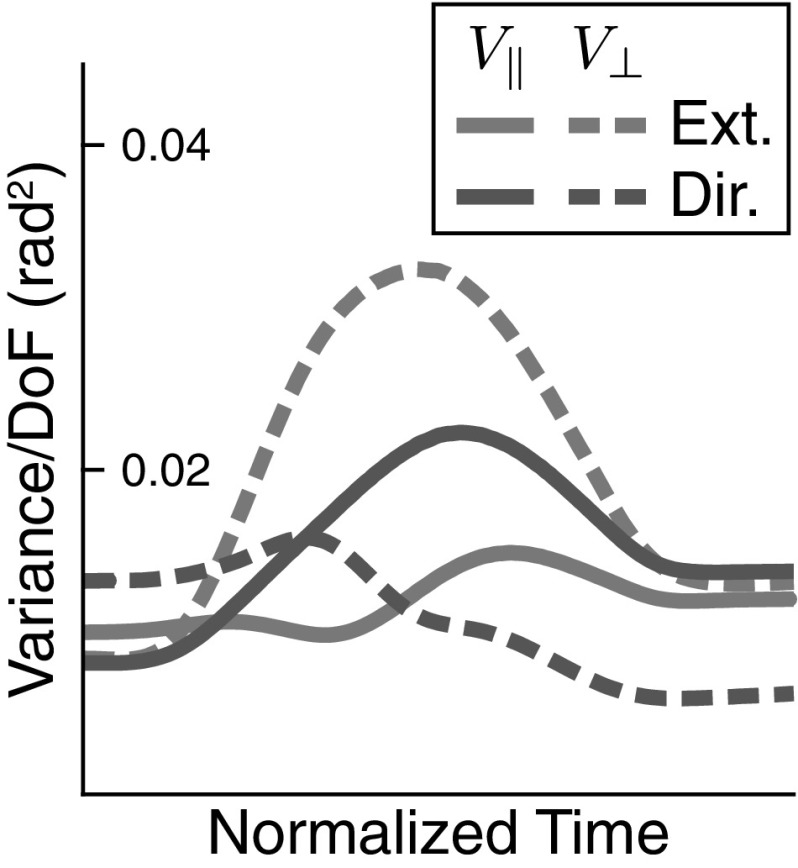
UCM structure of variance in simulations in which back-coupling within the null space has been replaced by damping

*Role of decoupling* In the space of virtual joint velocities, the two subspaces of the linearized UCM and its orthogonal complement are decoupled at each point in time, as described by Eq. 7. The descending timing signal is directed only into the space orthogonal to the UCM, and back-coupling is directed only into the UCM subspace. We can probe the role of this decoupling by replacing back-coupling by a damping term within that UCM subspace. This effectively provides stability within the UCM subspace. Figure 15 shows the structure of variance that emerges then. Muscle noise was eliminated to focus on the effect of the virtual trajectory dynamics alone. The UCM effect is effectively destroyed for the task variable “movement extent.” The UCM effect is strongly reduced for the task variable “movement direction”, persisting only in the second half of the movement.

## Discussion

We have shown how the UCM signature of variance may emerge from the processes of movement generation. These include (1) a process for generating the time course of the movement as a virtual end-effector trajectory, (2) a dynamical system that transforms the virtual end-effector trajectory into motor commands for the muscle-joint systems by decoupling those combinations of degrees of freedom that move the end-effector from those combinations that do not, (3) and a neural dynamics of muscular control that generate torques in response to descending motor commands and proprioceptive feedback. This process model had been developed earlier (Martin et al. 2009) to account for a wide range of kinematic properties of multi-degree-of-freedom reaching movements, including the self-motion observed in such systems as well as motor equivalence when a perturbation prevents the arm from reaching the configuration specified by descending commands. Motor equivalence, in particular, required an update of the motor command in response to proprioceptive signals in which the motor command “yields” to the current kinematic state of the limb, essentially the opposite of feedback control, which we named “back-coupling.” We also showed in that earlier work that the perfect inverse dynamics predicts too little self-motion, which supported the overall architecture of the proposed model that is based in virtual kinematic trajectories.

To account for the structure of variance in joint space, we systematically explored four sources of variability. We studied in detail the role of “muscle noise”, that is, of variance in the force levels generated by muscles given the same motor command. We also studied in detail the role of “neural noise”, that is, of variance in the motor commands that drive the muscle-joint systems. Random perturbations at the level of the timing of the end-effector virtual trajectory were examined, but had limited influence on the structure of variance in joint space. Finally, the variability of the initial joint configuration for each reaching movement was studied.

By comparing with experimental data, we were able to establish three accounts. (1) The overall UCM structure of joint configuration variance assessed by three features (Sect. 4.3, Fig. 10) was captured very well by the model. The account for the pattern across different movements and across time is perfectly adequate given the variation across participants. (2) The peak of variance in the direction perpendicular to the UCM around the time of peak end-effector velocity can largely be attributed to misalignment of different trials during time normalization and inhomogeneities in the time structure (Figs. 5, 6, and 7). (3) The UCM structure of joint configuration variance at the beginning of the movement was shown to be not the only source of UCM variance (Fig. 13). Given that UCM structure of variance is created by neural and muscle noise, the fact that this induced variance is preserved across trials when participants reset their limb to an initial end-effector position is natural in light of our earlier account for motor equivalence (Martin et al. 2009): When the arm is moved back to the starting position, the motor commands specified for each muscle-joint system are adjusted by back-coupling, enabling different initial postural states that reflect different motor-equivalent outcomes of that movement back to the starting position.

The process model of movement generation enabled us to explore the extent to which particular components of the model and particular sources of noise cause the UCM structure of variance. We identified three potential causes.

(1) The decoupling of the motor commands across the two subspaces parallel and orthogonal to the UCM induces a UCM signature in the structure of variance. This decoupling is formalized in the model by directing the descending virtual end-effector velocity to the subspace that is orthogonal to the UCM in Eq. 5, while the subspace parallel to the UCM receives no descending input (vector field zero except for back-coupling, see below). Note that the individual motor commands to different muscle-joint systems must be coupled in a particular way in order to achieve such decoupling.

The most direct evidence that decoupling causes a UCM signature of variance comes from the manipulation illustrated in Fig. 15 in which decoupling was perturbed by introducing a damping in the subspace parallel to the UCM. That damping stabilizes zero joint velocity in that subspace, disrupting the coordination among joints that decouples the two subspaces. Removing decoupling completely destroys the UCM effect with respect to the *movement extent* hypothesis, that is, the UCM induced by the position of the end-effector along its path to the target. It strongly reduces the UCM effect with respect to the *movement direction* hypothesis, the UCM induced by the deviation of the end-effector from its path to the target.

(2) The UCM structure of variance is caused, in part, by imperfect control, that is, failure to compensate perfectly for “muscle noise.” Specifically, the shape of the variance distribution in four-dimensional joint space that emerges from neural noise and decoupling alone is not sufficiently elongated along the UCM and compressed in the orthogonal directions, compared to the empirically observed structure (Figs. 11 and 12). In addition to neural noise, “muscle noise” generating random perturbation at the level of control is required for the model to capture that structure.

Muscles and their peripheral feedback control do not control the limb perfectly, so that it deviates from the joint configuration trajectory that the descending motor commands prescribe. We previously provided evidence for this claim by accounting for the sizable portion of self-motion observed in reaching with redundant degrees of freedom (Martin et al. 2009) and contrasting that observation to the very small amount of self-motion predicted with the model perfectly controls the descending commands. What we find here is that those random torque combinations that happen to affect the end-effector are resisted against more strongly than other random torque combinations that happen to lie in the UCM and do not affect the end-effector.

How does this happen? Some of the deviations from the descending motor command induced by muscle noise lie in the space orthogonal to the UMC. They are counteracted by the peripheral feedback loop that the muscle control model captures. Other deviations lie in the space parallel to the UCM. They are also counteracted by the peripheral muscle control. But at the same time, back-coupling updates the motor commands in this space toward the actual configuration. This leads to the virtual configuration following the random fluctuations induced by muscle noise to some degree, diminishing the extent to which muscle control counteracts those deviations.

(3) This role of back-coupling in causing the UCM structure of variance is highlighted by Fig. 14. With back-coupling in place, and under the influence of muscle noise alone (bottom left), the UCM component of variance increases over the course of the movement. When under the same conditions, back-coupling is eliminated, the UCM component of variance no longer increases significantly over the course of the movement. The remaining UCM effect is largely due to the structure of variance in the initial condition.

In response to neural noise, the opposite pattern emerges. Without back-coupling, because the vector field in the direction aligned with the UCM is zero, neural noise strongly drives variance in that direction. The observed increase in variance over time (Fig. 14, top right) reflects diffusion in that marginally stable direction in the space of motor commands. Back-coupling limits the extent to which this diffusion in the motor command is effective in driving variance of the joint configuration: As the motor command drifts away from the realized joint configuration, back-coupling pulls the motor command back to the realized configuration.

Together, we see how the coupling structure at the level of the motor commands and back-coupling combine to produce the UCM structure of variance. Variance within the motor commands is structured by the coupling that selectively stabilizes directions orthogonal to the UCM. Variance within the torque vectors that generate movement is selectively stabilized along directions orthogonal to the UCM by the peripheral feedback loops, while back-coupling partially removes that stabilizing effect within the UCM.

A related form of back-coupling has been postulated by Latash et al. (2005) to account for drifts within the UCM observed in isometric multi-finger force production tasks. That model shares with ours the structure of dynamic coupling among force generating units. That coupling structure generates compensatory changes in other units, when any given unit deviates from its mean state through “neural” noise conceived similarly as our neural noise. That model is strongly reduced compared to ours as the modeled isometric tasks are much simpler than point-to-point arm movements. The Jacobian is constant, for instance, no timing signal is required, and the muscle model can be omitted. As a result, this model does not address the other sources of the UCM structure of variance.

Another potential cause of the UCM structure of variance would be structure in the noise sources themselves. Goodman and Latash (2006) postulated, for instance, that the sources of noise that act within the UCM are larger in variance than the source of noise that act within the orthogonal complement. This makes it possible to generate the UCM structure of variance from a feed-forward control model. It may appear, however, that such an account merely shifts the problem to understanding where the difference in variance of the noise sources comes from.

Reaching, as we modeled it here, is ballistic in the sense that the overall movement parameters (direction, amplitude, and duration of the end-effector movement) are assumed fixed from the beginning of the movement. This is a common approximation that captures fast pointing or reaching movements quite well. Essentially, the time for feedback about the end-effector trajectory leading to updates of the motor plan is too short to be effective (but see Pruszynski et al. 2011). Such feedback is possible for slow movements, however. The other extreme limit case may be upright stance, in which typical sway frequencies of around 0.2 Hz imply a movement time of 5 s. Postural sway is dominated by feedback about the body’s (or the head’s) kinematic state in space. In such feedback systems, a fifth potential cause of the structure of variance has recently been described by Reimann and Schöner (2017). Assume that one degree of freedom (say the ankle in upright stance) causes a deviation of the relevant end-effector (here the body in space) from its set point. When that deviation is picked up by sensory systems, the feedback control system generates a compensatory movement plan that opposes that deviation. That motor plan is distributed to all the degrees of freedom in some form of inverse kinematics or kinetics. So, not only the degree of freedom originally causes the deviation, but also all other degrees of freedom are engaged in compensatory control, leading to a UCM structure of variance.

In summary, we have seen that a simplified, but still differentiated process model of movement generation enables us to pinpoint four different sources of the structure of variance: decoupling, motor noise, back-coupling, and transient suppression of variance. Such an analysis may be helpful in interpreting a host of experimental studies and may promote a research agenda of tracking changes of the structure of variance with learning (Kang et al. 2004) and development (Greve et al. 2017).

## Footnotes

1 Note that this is a different use of PCA than that made in a large number of other publications (e.g., Daffertshofer et al. 2004; Tresch et al. 2006). Here, we analyze strictly the variance across repetitions of the same movement at a fixed point in time and for a fixed movement. In those other approaches, variance across time and across tasks is often included or even made the main focus of analysis.
